# Effects of Hydrogen-Rich Water on Juvenile Largemouth Bass (*Micropterus salmoides*) Under Acute Low-Temperature Stress

**DOI:** 10.3390/antiox15060742

**Published:** 2026-06-11

**Authors:** Qianqian Xu, Haolin Wang, Xue Chen, Long Chen, Paini Xin, Hua Liu, Ying Yang

**Affiliations:** Guangdong Provincial Key Laboratory of Animal Molecular Design and Precise Breeding, School of Animal Science and Technology, Foshan University, Foshan 528225, China; xq_0405@126.com (Q.X.); 17513289671@163.com (H.W.); cv.246@foxmail.com (X.C.); 19852513197@163.com (L.C.); cyg5202017@outlook.com (P.X.); 13425990431@163.com (H.L.)

**Keywords:** hydrogen-rich water, *Micropterus salmoides*, acute low temperature, antioxidant capacity, intestinal microbiota

## Abstract

Hydrogen-rich water (HRW) is an aqueous solution containing dissolved molecular hydrogen. This study evaluated its effects on juvenile largemouth bass (*Micropterus salmoides*) under acute low-temperature stress. A total of 480 juveniles (2.4 ± 0.5 g) were randomly assigned to four groups: the control group was reared in standard water; the treatment groups were exposed to different hydrogen concentrations, specifically H1 (0.3 mg/L), H2 (0.5 mg/L), and H3 (0.9 mg/L). The fry were reared at 26 ± 0.5 °C for 30 days, followed by acute low-temperature stress (11 ± 0.5 °C) for 48 h. Samples were collected at 0, 8, 24, and 48 h. Results showed that after 30 days of HRW rearing, the final body weight (FBW), specific growth rate (SGR), and condition factor (CF) of the H1 group were significantly increased, while the H3 group only increased CF. No significant differences were observed in hepatopancreas somatic index (HSI) and survival rate (SR) among groups. Acute low-temperature stress induced liver and intestinal damage, which were alleviated in the H1 group. The H1 group exhibited significantly increased SOD, CAT, and GSH-Px activities in the liver, as well as CAT and SOD in the intestine and gills, while reducing MDA levels, thereby enhancing the antioxidant capacity. The H1 group significantly upregulated the antioxidant genes expression (*sod*, *cat*, and *gsh-px* mRNA levels) in the liver and gills but downregulated them in the intestine. 16S rDNA analysis revealed that HRW increased intestinal microbiota and the relative abundance of *Bacillota*. In conclusion, the H1 group significantly improved growth performance, mitigated acute low-temperature damage, enhanced antioxidant capacity, and increased the relative abundance of *Bacillota* in the intestines. This provides an innovative, safe, and effective solution for aquaculture industries confronting low-temperature challenges.

## 1. Introduction

In intensive aquaculture and during long-distance transport, acute low-temperature stress—triggered by cold snaps, seasonal shifts, or regional temperature variations—represents a primary driver of physiological stress, widespread disease outbreaks, and mortality in largemouth bass (*Micropterus salmoides*) fry [1]. Largemouth bass is native to North America and Canada and was introduced to Guangdong Province, China, in 1983, and are primarily cultivated in Guangdong, Zhejiang, and other regions [2]. Valued for its delicious taste, rapid growth rate, and short production cycle, it has become an important economically significant farmed fish in China’s freshwater aquaculture sector [2]. In 2023, China’s farmed production of largemouth bass reached 888,000 metric tons. However, as a warm-water species, its optimal growth temperature ranges from 26 to 29 °C [3,4]. When water temperatures drop to 12 °C, symptoms such as body tilting and reduced feeding activity appear; acute low-temperature stress at 10 °C can induce oxidative stress, leading to tissue damage and an imbalance in the antioxidant system [5,6]. Therefore, enhancing largemouth bass tolerance to acute low-temperature stress is critical to ensuring stable, sustainable production within the industry.

From a molecular perspective, a low temperature induces excessive accumulation of reactive oxygen species (ROS), which oxidize cellular lipids, proteins, and DNA, causing oxidative stress, apoptosis, and tissue dysfunction [7]. Although fish can mount a defense by upregulating antioxidant enzyme genes (such as *sod* and *cat*), under conditions of rapid cooling (temperature decline > 6 °C/h), the endogenous antioxidant system is insufficient to counteract ROS. This leads to metabolic suppression, intestinal microbiota dysbiosis, and, in severe cases, mortality [8,9,10]. Conventional strategies to mitigate low-temperature stress mainly depend on chemical anesthesia and the supplementation of antioxidants (e.g., vitamin C). However, traditional antioxidants have significant limitations. Consequently, identifying green, safe, and low-cost exogenous antioxidants has become an urgent need to enhance fish resistance to low temperature.

Hydrogen-rich water (HRW) is an aqueous solution containing dissolved molecular hydrogen, typically produced through electrolysis or chemical reactions [11]. As a novel selective antioxidant, HRW reduces ROS accumulation by inhibiting NADPH oxidase activity and preventing mitochondrial damage [12]. HRW selectively neutralizes •OH and ONOO^−^ while activating the kelch-like ECH-associated protein 1 (Keap1)—nuclear factor erythroid 2-related factor 2 (Nrf2) signaling pathway, thereby enhancing the activities of SOD, CAT, and GSH-Px to alleviate oxidative stress [12,13]. Recent research on the application of HRW in aquatic animals has begun to attract attention. For instance, HRW (179.65 ± 31.95 ppb) significantly improves the survival rate (SR) and growth performance of largemouth bass, promotes intestinal digestion, and increases the abundance of beneficial intestinal bacteria [14]. Furthermore, HRW mitigates the toxic effects of microplastics (MP) on rainbow trout (*Oncorhynchus mykiss*) through multiple mechanisms, including alleviation of oxidative stress, reduction in DNA damage, inhibition of apoptosis, and neuroprotective [15]. However, existing studies have investigated pathogen infection or growth promotion under normal temperature conditions, with limited exploration of HRW intervention in acute low-temperature stress models. Whether HRW can alleviate acute low-temperature-induced oxidative damage in largemouth bass through Keap1-Nrf2 pathway activation remains to be elucidated.

Therefore, this study subjected juvenile largemouth bass to acute low-temperature stress (11 ± 0.5 °C)—a temperature below the lower critical thermal limit that reliably induces oxidative stress—to investigate the regulatory effects of different concentrations of HRW on antioxidant capacity, intestinal microbial community structure, and gene expression in the Keap1-Nrf2 signaling pathway in the liver, intestine, and gill tissues of the juveniles. This study aims to elucidate the effects of HRW in mitigating acute low-temperature damage in fish, thereby providing a theoretical basis for low-temperature stress prevention and control in aquaculture.

## 2. Materials and Methods

### 2.1. Experimental Design

All experimental procedures were approved by the Experimental Animal Ethics Committee of Foshan University (FOSU196804).

Juvenile largemouth bass (2.4 ± 0.5 g) were sourced from a commercial farm in Foshan, China, and acclimated in 400 L plastic tanks at 26 ± 0.5 °C for one week. A total of 480 largemouth bass fry (2.4 ± 0.5 g) were randomly divided into four groups: a control group in standard water, and three HRW treatment groups (H1: 0.3 mg/L; H2: 0.5 mg/L; H3: 0.9 mg/L dissolved hydrogen). Each group had three replicates, with 40 fish per replicate. Hydrogen was continuously supplied daily for a 30-day rearing period. The hydrogen in the HRW existed as physically dissolved molecular hydrogen, produced by electrolysis of water using a PEM proton exchange membrane device (Guangdong Kawolo Small Appliances Co., Ltd. (Foshan, China)). Hydrogen concentration in the water was measured using a TRUSTLEX ENH-2000 (TRUSTLEX, Osaka, Japan). During the experiment, fish were fed twice daily at 7:00 and 17:00. They were fed commercial feed ad libitum. The water temperature was maintained at 26 ± 0.5 °C, and dissolved oxygen at 6.5 ± 0.2 mg/L. At the end of the rearing period, fish were fasted for 24 h prior to measurement of final body weight (FBW), body length, and SR.

Subsequently, an acute low-temperature stress test was then conducted. The water temperature was lowered from 26 ± 0.5 °C to 11 ± 0.5 °C at a rate of 2 °C/h. This cooling rate was based on recommended parameters for cold storage of largemouth bass and waterless preservation of spotted knifejaw (*Oplegnathus punctatus*) [6,16]. The cooling process was achieved by adding pre-chilled sealed ice packs and continuous aeration. Water temperature was monitored every 15 min during cooling to ensure the temperature variation remained within 0.5 °C. Once the water temperature reached 11 ± 0.5 °C and stabilized for 30 min, this was recorded as 0 h, and the 48 h acute low-temperature stress experiment began. Feeding was suspended during acute low-temperature stress. No mortality was observed in any group of largemouth bass fry during the experiment.

### 2.2. Sample Collection

Samples were collected at four time points (0, 8, 24, and 48 h) following acute low-temperature stress. At each sampling time point, juvenile largemouth bass from different individuals were used; specifically, 15 largemouth bass were randomly selected from each group (including replicate groups) at each time point, and the sampled fish were not returned to their original tanks. Under eugenol (50 mg/L) anesthesia, blood was collected from the caudal vein using a sterile 1.0 mL syringe. Blood samples were collected in 2 mL enzyme-free Eppendorf (EP) tubes, allowed to stand at 4 °C for 2 h, and then centrifuged (3500 r/min, 4 °C, 10 min). The supernatant serum was aspirated and transferred to new 1.5 mL enzyme-free EP tubes for storage at −80 °C. Immediately after blood collection, liver, intestine, and gill tissue samples were collected into 2 mL enzyme-inactive EP tubes, rapidly frozen in liquid nitrogen, and transferred to −80 °C for storage, to be used for the determination of antioxidant enzyme activity and relative gene expression. For gill tissue, the first gill filament on the right side was collected. Concurrently, at each time point, three fish were randomly selected from each group, and intact liver and intestinal tissues were collected and fixed in 4% paraformaldehyde for histopathological examination. At the 24 h time point, an additional 3 fish were selected from each replicate in each group. Intestinal contents and intestinal tissues were collected under sterile conditions, rapidly frozen in liquid nitrogen, and stored at −80 °C for 16S rDNA sequencing analysis of intestinal microbiota.

### 2.3. Growth Performance

The calculation formula is as follows:

Specific growth rate (SGR) = 100 × [(ln final weight − ln initial weight)/Rearing period]

Hepatopancreas somatic index (HSI) = 100 × (Liver weight/Body weight)

Condition factor (CF) = 100 × [final weight/(body length)^3^]

Survival rate (SR) = 100 × (number of surviving fish/initial number of fish)

### 2.4. Histological Analysis

From each group, the livers and intestines of two randomly selected fish were taken, rinsed with saline, and immediately immersed in a 4% paraformaldehyde solution. After fixation, the tissues were rinsed under running water, dehydrated in a series of ethanol solutions, cleared with xylene, and embedded in paraffin. Sections 7 μm thick were cut from the embedded samples, stained with hematoxylin and eosin (H&E), and finally mounted with neutral resin. Observations were made under an optical microscope (Guangzhou, China; Guangzhou Daoyi Science and Technology Co., Ltd.).

For each treatment group and time point, the villus height (VH) (defined as the vertical distance from the tip of the villus to the opening of the crypt) was measured in 3 fish. From the intestinal sections of each fish, 5 morphologically intact villi with straight orientation were randomly selected.

### 2.5. Biochemical Analysis

Serum ALT and AST levels were measured using commercial kits (Nanjing Jiancheng Biotechnology Research Institute, Nanjing, China). The kit lot numbers were C009-2-1 and C010-2-1, respectively.

Liver, intestinal, and gill tissue samples were homogenized at low temperatures in a 1:9 tissue-to-saline ratio, and the supernatant was collected for analysis. Total protein (TP), malondialdehyde (MDA), superoxide dismutase (SOD), catalase (CAT), and glutathione peroxidase (GSH-Px) levels were measured using kits provided by the same manufacturer. The kit lot numbers were: A045-2-2, A003-1-2, A001-3-2, A007-1-1, and A005-1-2.

### 2.6. RNA Extraction and qPCR Amplification

Liver, intestinal, and gill tissue samples (50–100 mg) collected at each time point were ground into a powder, transferred to 1.5 mL of sterile EP tubes, mixed with 1 mL of TransZol Up and 0.2 mL of RNA Extraction Agent, and total RNA was extracted using the TransZol Up Plus RNA Kit (Beijing, China: Beijing Quanshijin Biotechnology Co., Ltd.). After RNA extraction, the concentration and quality of the RNA samples were analyzed using a Q5000 (Quawell, San Jose, CA, USA) and 1% agarose gel electrophoresis. cDNA was synthesized using the TransScript^®^ One-Step gDNA Removal and cDNA Synthesis SuperMix Kit (Beijing, China; Beijing Quanshijin Biotechnology Co., Ltd.) and stored at −20 °C. qPCR amplification was performed on the QuantStudio 5 system using the PerfectStart Green qPCR SuperMix Kit (Beijing, China: Beijing Quanshijin Biotechnology Co., Ltd.). Primer sequences are shown in Table 1. *β-actin* was used as an internal control, and relative gene expression was calculated using the 2^−ΔΔCt^ method.

### 2.7. 16S rDNA Sequencing

#### 2.7.1. Total Bacterial DNA Extraction and PCR Amplification

Samples were collected 24 h after acute low-temperature stress. Intestinal contents and tissues were aseptically collected, snap-frozen in liquid nitrogen, and stored at −80 °C. A total of 16 samples—four from each treatment group—were analyzed via sequencing. Microbial community structure and diversity were analyzed using high-throughput sequencing technology. Total genomic DNA was extracted from all intestinal and intestinal contents samples, and DNA quantification was performed using Qubit (Invitrogen, Carlsbad, CA, USA). Subsequently, PCR amplification of the 16S rDNA V3–V4 region was performed using the primers (341F: 5′-CCTACGGGNGGCWGCAG-3′; 805R: 5′-GACTACHVGGGTATCTAATCC-3′) to amplify the 16S rDNA V3–V4 region. The reaction volume was 25 μL. The PCR amplification program was as follows: 30 s pre-denaturation at 98 °C; 10 s denaturation at 98 °C, 30 s annealing at 50 °C, and 45 s extension at 72 °C, for 32 cycles; followed by a final extension at 72 °C for 10 min. PCR products were purified using AMPure XP beads (Beckman Coulter Genomics, Danvers, MA, USA) and quantified with Qubit (Invitrogen, USA). The purified PCR products were quality-assessed and used to construct libraries using the Illumina (KapaBiosciences, Woburn, MA, USA) library quantification kit.

#### 2.7.2. High-Throughput Sequencing

After the library passed quality control, paired-end sequencing was performed using a high-throughput sequencing kit (G99 App-D FCL PE300; Cat. No.: 940-001716-00; MGI Tech Co., Ltd., Shenzhen, China) on a DNBSEQ-G99 sequencer. All analyses were performed by Lianchuan Biotechnology Co., Ltd. (Hangzhou, China). The data supporting the findings of this study are openly available in the NCBI Sequence Read Archive (SRA) at https://dataview.ncbi.nlm.nih.gov/object/PRJNA1467364 (accessed on 19 May 2026), accession number PRJNA1467364.

#### 2.7.3. Bioinformatics Analysis

After sample-specific separation of the paired-end reads obtained from sequencing, the reads were first subjected to quality control and filtering based on sequencing quality. Alpha-diversity indices, including the Ace, Chao1, Shannon, and Simpson indices, were calculated based on the operational taxonomic unit (OTU) table to evaluate microbial community richness and diversity.

### 2.8. Statistical Analyses

All data are presented as mean ± SE. The statistical analysis in this study followed the method described by Liu et al. [17] and was performed using SPSS 27.0 with Duncan’s multiple range test. Statistical significance was set at *p* < 0.05. Graphs were generated using Prism 10 (GraphPad Software, Inc., Solana Beach, CA, USA).

## 3. Results

### 3.1. Fish Growth Performance Indicators

Growth performance is shown in Table 2. Results showed that FBW, SGR, and CF in H1 group were significantly higher than those in the control group (*p* < 0.05). Additionally, CF in the H3 group was significantly higher than that in the control group (*p* < 0.05). There were no significant differences in HSI and SR among the groups (*p* > 0.05).

### 3.2. Histopathological Analysis of Liver and Intestinal Tissue

**Figure 1:**
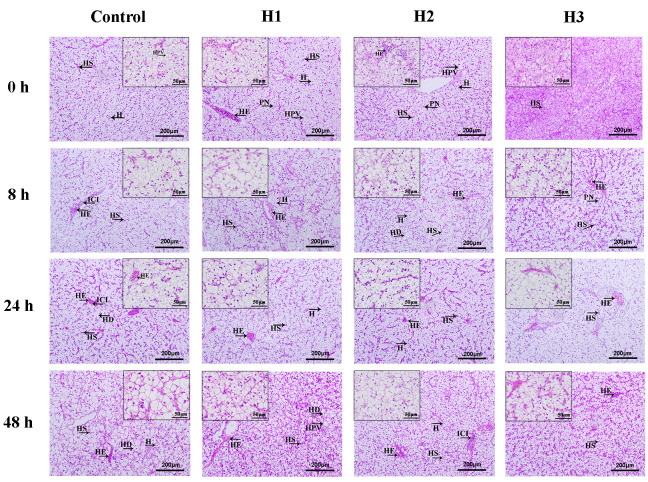
Effects of HRW on the histological structure of largemouth bass liver tissues. HS, hepatic sinusoid; H, hepatocyte; HE, hemorrhage; ICI, inflammatory cell infiltration; PN, cellular peripheral nucleus; HD, hydropic degeneration; HPV, hepatocellular vacuolation.

Figure 1 shows liver tissue images. In the control group, hepatocyte edema, vacuolation, and nuclear displacement appeared at 8 and 24 h, accompanied by hemorrhage and inflammatory cell infiltration; hepatocyte swelling worsened at 48 h. In contrast, the H1 and H2 groups exhibited mild hemorrhage at 8 h with fewer vacuoles, clearer hepatocyte boundaries, and no inflammatory cells. The H2 group showed inflammatory cells at 48 h, while the H3 group demonstrated inflammatory cells at 24 h.

**Figure 2:**
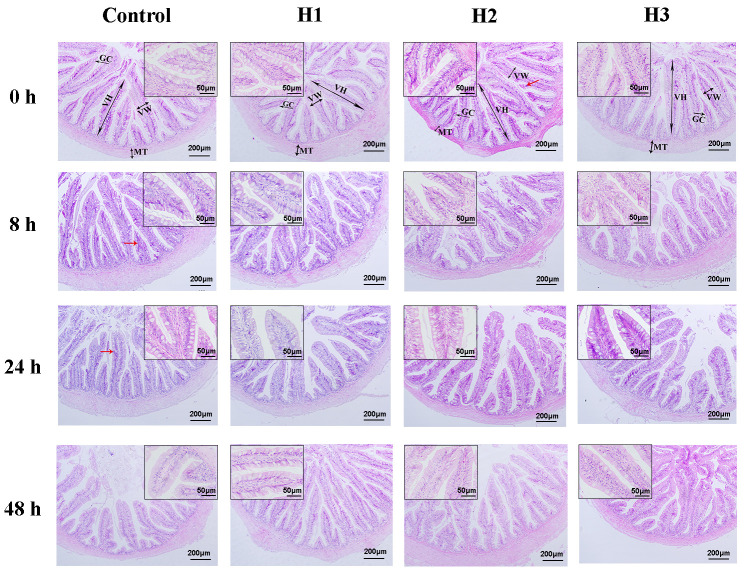
Effects of HRW on intestinal tissue structure in largemouth bass. VH, villi length; VW, villi width; GC, goblet cell; MT, muscular thickness; red arrow, inflammatory cell infiltration (ICI).

Figure 2 shows intestinal tissue images. The control group exhibited inflammatory cell infiltration (ICI) (red arrows) and reduced goblet cells at 8 and 24 h. The H1 group demonstrated decreased inflammatory cells at all time points with well-preserved villus structure. The H2 group showed villus damage at 8 h, gradually recovering at 24 and 48 h. At 48 h, the H3 group exhibited disorganized intestinal villus arrangement.

Intestinal villus length is summarized in Table 3. At 24 and 48 h, villus length in the control group was significantly shorter than pre-stress levels (*p* < 0.05). Compared to the control group, villus length in the HRW group was significantly shorter at 8 h but significantly longer at 24 and 48 h (*p* < 0.05).

### 3.3. Serum ALT and AST Activities

**Figure 3:**
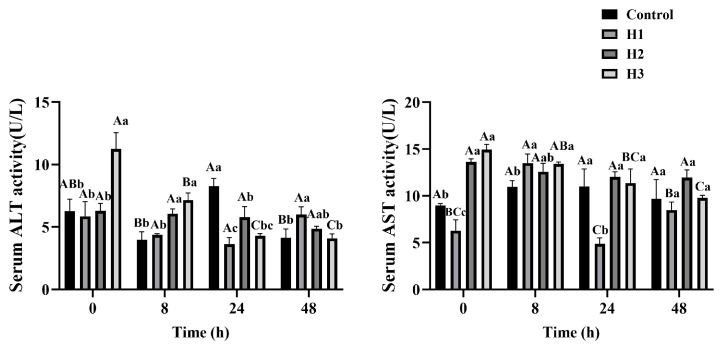
Effects of HRW on serum biochemical indicators of largemouth bass. Data are presented as mean ± SE, n = 6. Different capital letters indicate significant differences between different time points within the same group (*p* < 0.05), different lowercase letters indicate significant differences between different groups at the same time point (*p* < 0.05). ALT, alanine aminotransferase; AST, aspartate aminotransferase.

Figure 3 shows that the serum ALT level in the control group increased initially and then decreased within 8 to 48 h, peaking at 24 h (*p* < 0.05). The serum AST level in the control group did not show any significant change over the course of stress (*p* > 0.05). At 24 h, the serum ALT levels in the HRW treatment groups (H1, H2, and H3 groups) were significantly lower than that in the control group (*p* < 0.05), while only the H1 group had a significantly lower AST level than the control group (*p* = 0.003).

### 3.4. Antioxidant-Related Enzyme Activities and Gene Expressions in the Liver

**Figure 4:**
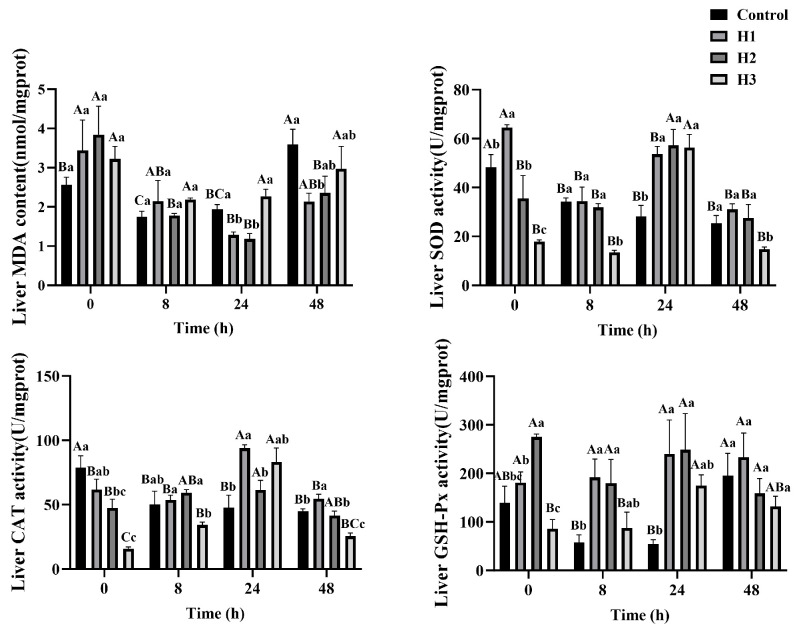
Effects of HRW on biochemical indicators in the liver of largemouth bass. Data are expressed as mean ± SE, n = 6. Different capital letters indicate significant differences between different time points within the same group (*p* < 0.05), different lowercase letters indicate significant differences between different groups at the same time point (*p* < 0.05). MDA, malondialdehyde; SOD, superoxide dismutase; CAT, catalase; GSH-Px, glutathione peroxidase.

The results for liver antioxidant enzyme activity are shown in Figure 4. Acute low-temperature stress led to a decrease in liver antioxidant enzyme activity in the control group, accompanied by elevated MDA levels, whereas the H1 and H2 groups alleviated oxidative damage. In the control group, 8 h after stress, both SOD and CAT activities were significantly lower than pre-stress levels (*p* < 0.05); MDA levels were significantly lower at 8 h compared to pre-stress levels (*p* < 0.05), but significantly increased and reached a peak at 48 h (*p* < 0.05), indicating that prolonged stress may lead to exacerbated liver damage. Compared with the control group, 24 h after stress, MDA levels in the H1 and H2 groups were significantly reduced, while SOD and GSH-Px activities were significantly increased (*p* < 0.05); additionally, CAT activity in the H1 group was significantly higher than that in the control group (*p* = 0.023). At 48 h post-stress, CAT activity in the H1 group had returned to baseline levels; in contrast, SOD and CAT activities in the H3 group were significantly lower than those in the control group (*p* < 0.05), suggesting that high hydrogen concentrations may have an adverse effect on the antioxidant system.

**Figure 5:**
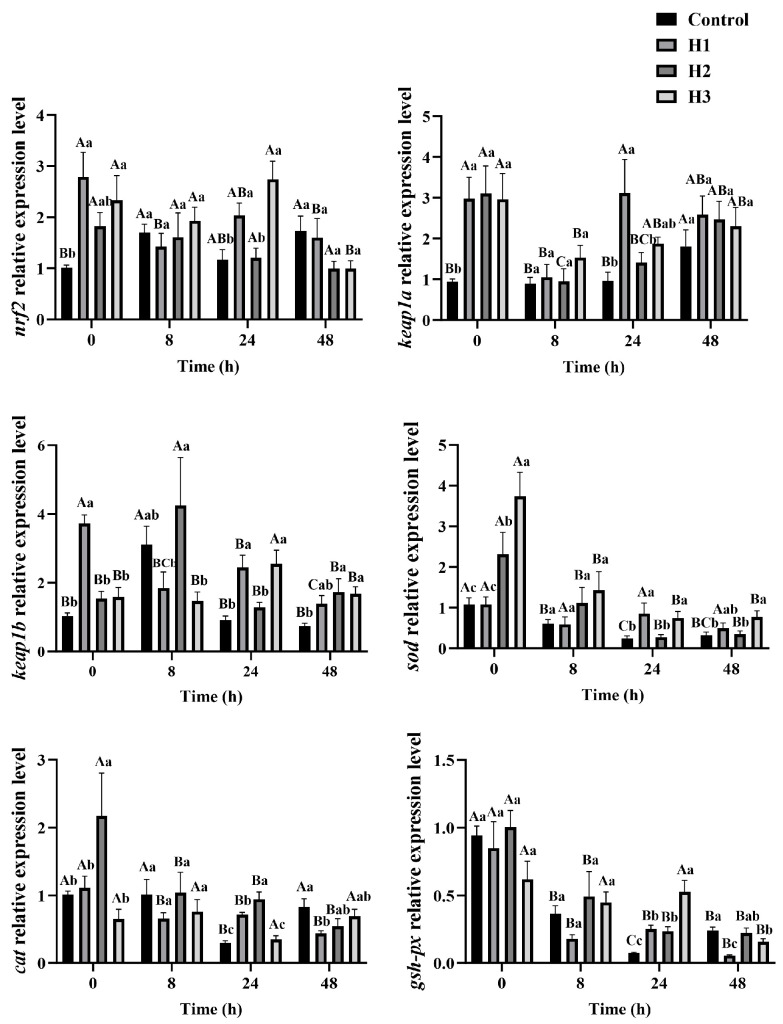
The effect of HRW on the mRNA expression in the liver of largemouth bass. Data are presented as mean ± SE, n = 9. Different capital letters indicate significant differences between different time points within the same group (*p* < 0.05), different lowercase letters indicate significant differences between different groups at the same time point (*p* < 0.05). The *nrf2*, nuclear factor erythroid 2-related factor 2; *keap1a*, kelch-like ECH-associated protein 1a; *keap1b*, kelch-like ECH-associated protein 1b; *sod*, superoxide dismutase; *cat*, catalase; *gsh-px*, glutathione peroxidase.

The results of liver gene expression are shown in Figure 5. In the control group, *keap1b* mRNA levels were significantly upregulated at 8 h of stress (*p* < 0.05) and returned to baseline levels by 24 h; at 48 h of stress, the *nrf2* and *keap1a* mRNA levels were significantly upregulated (*p* < 0.05), while *sod* and *gsh-px* mRNA levels were significantly downregulated (*p* < 0.05), indicating that prolonged stress may suppress the liver’s antioxidant defense capacity. Compared with the control group, at 24 h of stress, the *nrf2*, *keap1a*, *keap1b*, *sod*, *cat*, and *gsh-px* mRNA levels were significantly elevated in H1 group (*p* < 0.05); additionally, the *cat* and *gsh-px* mRNA levels were also significantly upregulated in H2 group (*p* < 0.05); at 48 h post-stress, the *cat* and *gsh-px* mRNA levels in the H1 group were significantly downregulated (*p* < 0.05).

### 3.5. Antioxidant-Related Enzyme Activities and Gene Expressions in the Intestine

**Figure 6:**
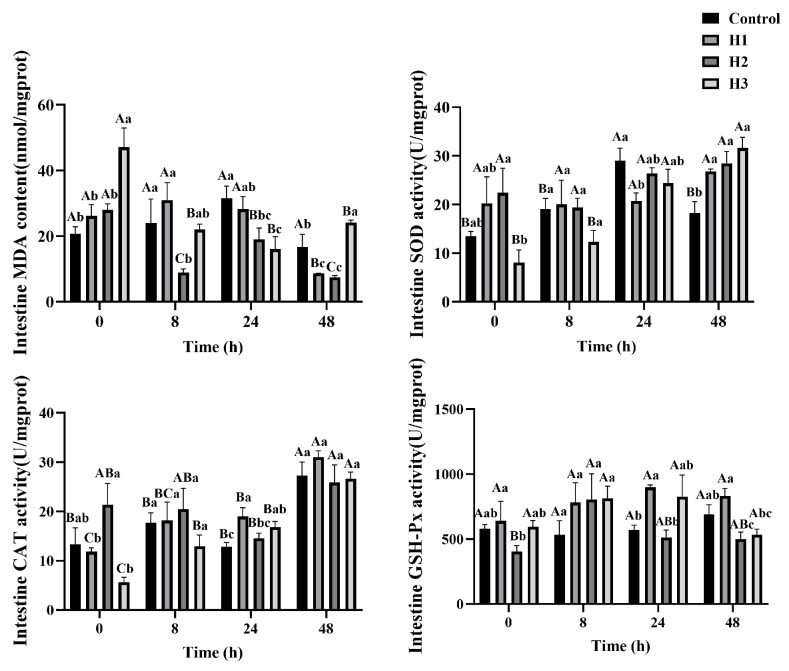
Effects of HRW on intestinal biochemical parameters in largemouth bass. Data are expressed as mean ± SE, n = 6. Different capital letters indicate significant differences between different time points within the same group (*p* < 0.05), different lowercase letters indicate significant differences between different groups at the same time point (*p* < 0.05).

The activities of antioxidant-related enzymes in the intestine are shown in Figure 6. Compared with the liver, the antioxidant response in the intestine exhibited different characteristics. In the control group, MDA levels and GSH-Px activity did not change significantly throughout the stress process (*p* > 0.05). SOD activity peaked at 24 h post-stress, while CAT activity peaked at 48 h (*p* < 0.05). Compared with the control group, CAT and GSH-Px activities were significantly elevated in the H1 group at 24 h (*p* < 0.01), and MDA levels were significantly reduced in the H2 and H3 groups (*p* < 0.05). By 48 h of stress, MDA levels in both the H1 and H2 groups were significantly lower than those in the control group and pre-stress levels (*p* < 0.01), and SOD activity in the HRW group was significantly higher than that in the control group (*p* < 0.01). This indicates that HRW treatment helps alleviate intestinal oxidative damage and enhances antioxidant capacity.

**Figure 7:**
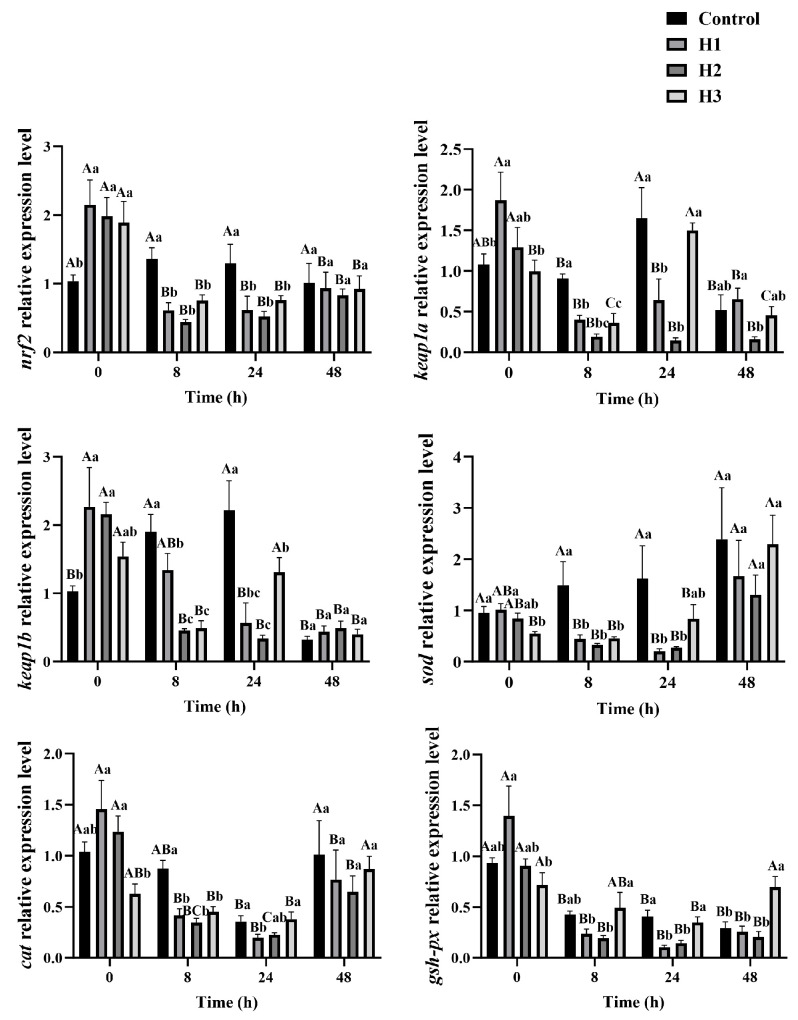
Effects of HRW on mRNA expression in the intestines of largemouth bass. Data are presented as mean ± SE, n = 9. Different capital letters indicate significant differences between different time points within the same group (*p* < 0.05), different lowercase letters indicate significant differences between different groups at the same time point (*p* < 0.05).

The results of intestinal gene expression are shown in Figure 7. The stress response patterns of intestinal antioxidant-related genes to acute low-temperature stress differed significantly from those in the liver. In the control group, *cat* and *gsh-px* mRNA levels were significantly reduced at 24 h post-stress (*p* < 0.05); *keap1b* mRNA levels were significantly elevated at 8 h and 24 h (*p* < 0.05) and returned to baseline levels at 48 h; *nrf2* mRNA levels did not change significantly with prolonged stress duration (*p* > 0.05). Compared with the control group, at 8 h post-stress, *nrf2*, *keap1a*, *keap1b*, *sod*, and *cat* mRNA levels were significantly downregulated in the HRW group (*p* < 0.05); at 24 h post-stress, *nrf2* and *keap1b* mRNA levels in the HRW groups were significantly downregulated (*p* < 0.05), with *keap1a*, *sod*, and *gsh-px* mRNA levels significantly downregulated in both the H1 and H2 groups (*p* < 0.05); additionally, only the *cat* mRNA level in the H1 group was significantly downregulated (*p* < 0.05). By 48 h, compared with pre-stress levels, *nrf2*, *keap1a*, and *keap1b* mRNA levels were significantly downregulated in the HRW groups, while *cat* and *gsh-px* mRNA levels were also significantly downregulated in both the H1 and H2 groups (*p* < 0.05). Combined with the enzyme activity results, it can be seen that changes in antioxidant enzyme activity in the intestine do not fully align with the trends in mRNA expression, suggesting that the intestinal antioxidant response may be influenced by tissue-specific regulation.

### 3.6. Antioxidant-Related Enzyme Activities and Gene Expressions in the Gill

**Figure 8:**
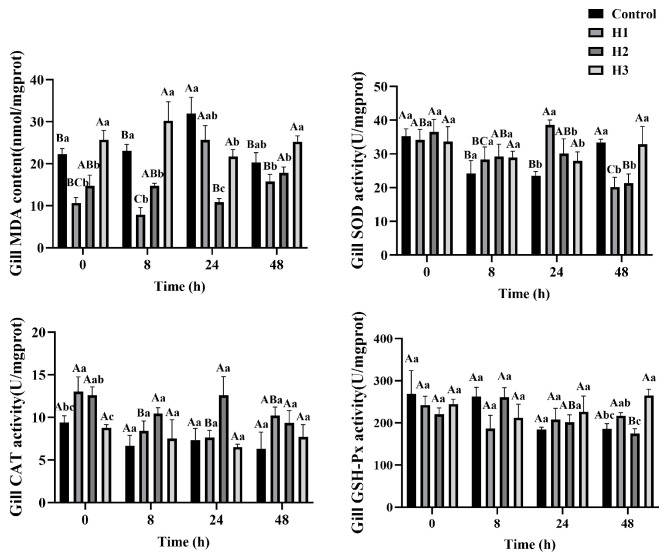
Effects of HRW on gills biochemical parameters in largemouth bass. Data are presented as mean ± SE, n = 6. Different capital letters indicate significant differences between different time points within the same group (*p* < 0.05), different lowercase letters indicate significant differences between different groups at the same time point (*p* < 0.05).

The activities of gill antioxidant-related enzymes are shown in Figure 8. In the control group, compared with pre-stress levels, MDA content significantly increased 24 h after stress (*p* < 0.05), while SOD activity significantly decreased at 8 h and 24 h after stress (*p* < 0.05); by 48 h, MDA and SOD had returned to baseline levels; CAT and GSH-Px activities in the control group did not show significant changes over the course of stress (*p* > 0.05). Compared with the control group, MDA levels were significantly reduced in the H1 and H2 groups at 8 h post-stress (*p* < 0.01); at 24 h post-stress, MDA levels were significantly reduced in the H2 and H3 groups, and SOD activity was significantly increased in the H1 group (*p* < 0.001). By 48 h, SOD activity in the H1 and H2 groups had significantly decreased, while GSH-Px activity in the H3 group had significantly increased (*p* < 0.05).

**Figure 9:**
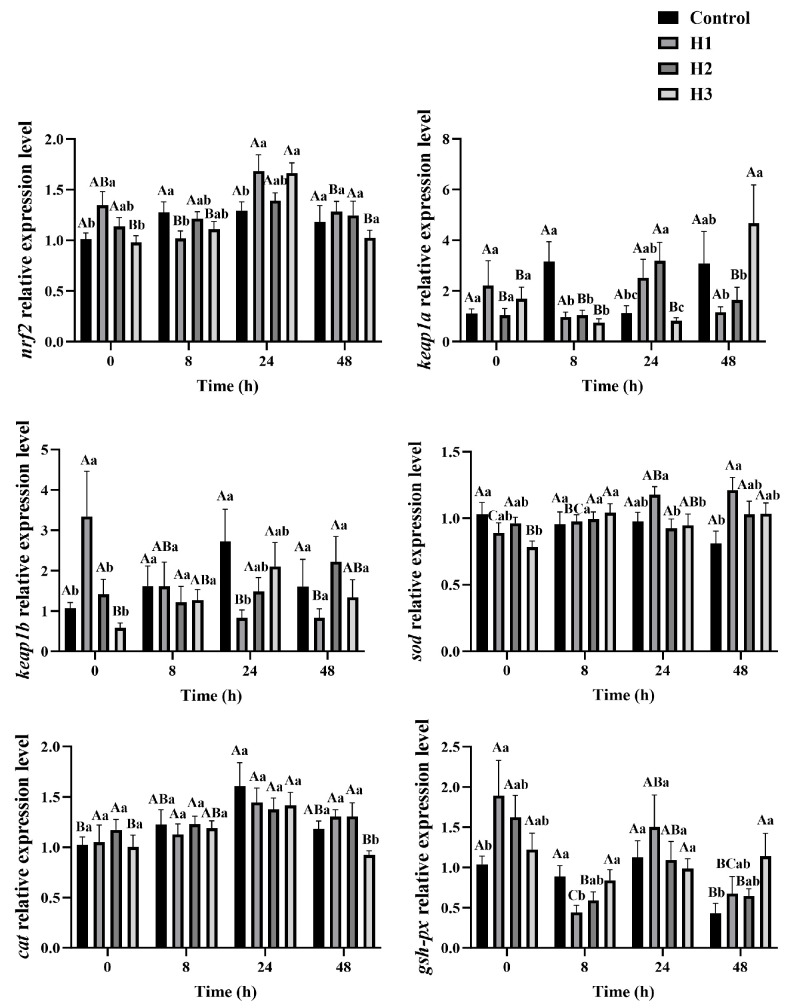
Effects of HRW on mRNA expression in the gills of largemouth bass. Data are presented as mean ± SE, n = 9. Different capital letters indicate significant differences between different time points within the same group (*p* < 0.05), different lowercase letters indicate significant differences between different groups at the same time point (*p* < 0.05).

The results of gill gene expression are shown in Figure 9. Compared with pre-stress levels, the control group exhibited significantly elevated *cat* mRNA levels at 24 h post-stress and significantly reduced *gsh-px* mRNA levels at 48 h (*p* < 0.05). No significant changes were observed in the control group for *nrf2*, *keap1a*, *keap1b*, and *sod* mRNA levels (*p* > 0.05). Compared with the control group: At 8 h post-stress, *keap1a* mRNA levels were significantly reduced in HRW groups (*p* < 0.05), and *gsh-px* mRNA levels were also significantly reduced in the H1 group (*p* < 0.05). At 24 h, *nrf2* mRNA levels were significantly upregulated in the H1 group, while *keap1b* mRNA levels were significantly reduced (*p* < 0.05). At 48 h, the H3 group showed significantly increased *gsh-px* and decreased *cat* expression (*p* < 0.05), while the H1 group exhibited significantly increased *sod* mRNA levels (*p* < 0.05).

### 3.7. Intestinal Microbiota

**Figure 10:**
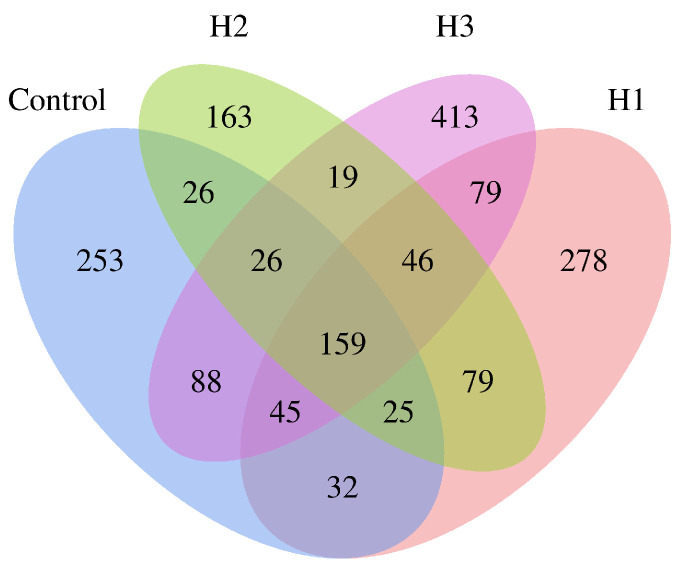
Venn diagram of OTUs of intestinal microbiota in largemouth bass at 24 h of acute low-temperature.

The diversity analysis of the intestinal microbiota of largemouth bass at 24 h is as follows. The Venn diagram analysis is shown in Figure 10. A total of 159 OTUs overlapped among the four groups, with control, H1, H2, and H3 having 253, 278, 163, and 413 unique OTUs, respectively. Table 4 indicates that Chao1, Shannon, and Simpson indices showed no significant differences (*p* > 0.05). However, the Ace index for H3 was significantly higher than that for control (*p* < 0.05).

**Figure 11:**
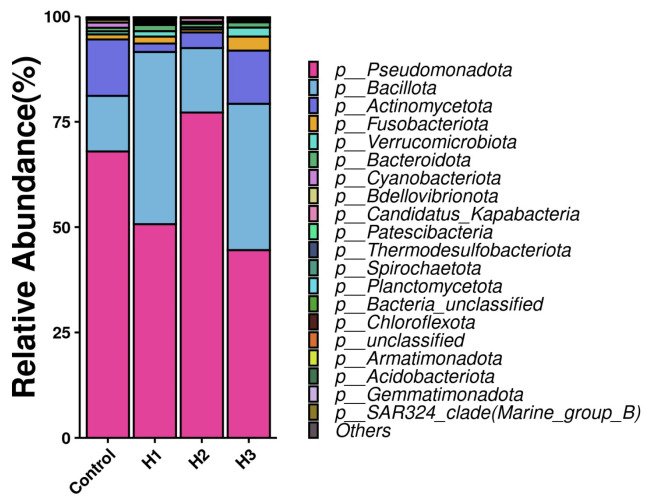
Abundance of intestinal microbial communities at the phylum level in largemouth bass after 24 h of acute low temperature.

Figure 11 shows that the dominant groups in the microbial community at the phylum level are the *Pseudomonadota*, *Bacillota,* and *Actinomycetota*. In contrast, the abundance of *Pseudomonadota* in the H1 and H3 groups significantly decreased while it increased in the H2 group (*p* < 0.05); the abundance of *Actinomycetota* in both the H1 and H2 groups significantly decreased (*p* < 0.05). The abundance of *Bacillota* in the H1 and H3 groups significantly increased (*p* < 0.05).

**Figure 12:**
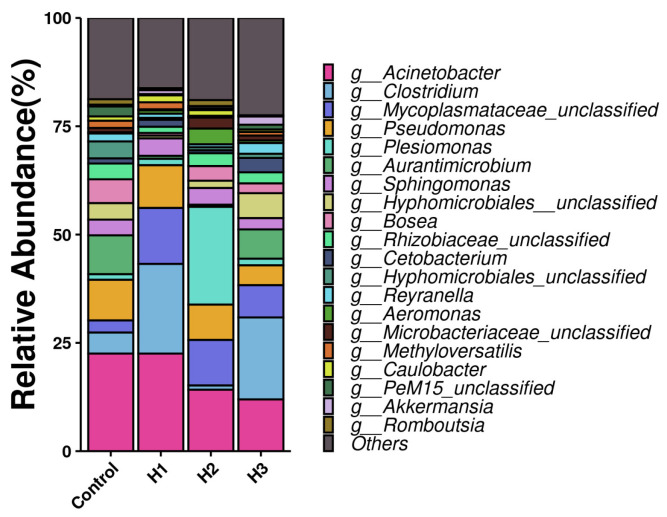
Abundance of intestinal microbial communities at the genus level in largemouth bass after 24 h of acute low temperature.

Figure 12 shows that the dominant community structure at the genus level mainly consists of *Acinetobacter*, *Clostridium*, *Mycoplasmataceae_unclassified*, *Pseudomonas*, *Plesiomonas,* and *Aurantimicrobium*. In H1 group, the relative abundances of *Cetobacterium* and *Clostridium* increased. In H2 and H3 groups, *Acinetobacter* abundance decreased significantly, while *Aeromonas* abundance increased significantly (*p* < 0.05). In the H3 group, the abundance of the *Akkermansia* significantly increased (*p* < 0.05).

## 4. Discussion

The results of this study indicate that HRW treatment significantly improved the growth performance of juvenile largemouth bass; in particular, the FBW, SGR, and CF values in H1 group were significantly higher than those in the control group, suggesting that HRW at an appropriate concentration may serve as a safe and reliable water source for aquaculture [14]. In aquatic ecosystems, hydrogen acts as an exogenous physiological regulator; its unique characteristics include an extremely low molecular weight and high membrane permeability [13]. This stands in stark contrast to terrestrial mammals, which ingest HRW via the digestive tract. In an HRW environment, fish can efficiently exchange gases and absorb them through osmosis via the microvasculature of the gill filaments, the skin, and the intestinal mucosa [18], enabling rapid systemic physiological responses even at lower concentrations. Furthermore, the significant increase in CF in H3 group further confirms that HRW has a positive effect on the robustness of the fish. Furthermore, there were no significant differences in HSI and SR among the groups, suggesting that while HRW promotes growth, it likely does not cause significant pathological damage to the liver, thereby enhancing the aquaculture efficiency of juvenile largemouth bass while ensuring the health of the fish.

Temperature is a key environmental factor regulating physiological processes such as growth and development in fish. Acute low-temperature exposure leads to hepatic hemorrhage, cellular edema, and vacuolization, accompanied by a rise followed by a decline in serum ALT levels between 8 and 48 h, consistent with previous studies [19,20], confirming that acute low-temperature stress causes structural damage to hepatocytes. The elevation in serum ALT without significant changes in AST suggests that liver damage is primarily localized to the cell membrane and cytoplasm, and may not yet have caused mitochondrial damage [21]. HRW is capable of protecting cellular structures; furthermore, ALT and AST levels in the H1 group were significantly lower than those in the control group 24 h after stress, confirming that HRW can stabilize biological membrane structures and alleviate liver damage [22]. Low temperature can also disrupt intestinal tissue integrity [23]. Acute low-temperature stress leads to intestinal villus atrophy and a reduction in goblet cell numbers, consistent with findings in freshwater drum (*Aplodinotus grunniens*) [24] and hybrid yellow catfish (*Trachinotus ovatus*) [25], confirming that acute low-temperature stress disrupts intestinal structure. In contrast, HRW treatment (particularly in the H1 group) improved intestinal structural damage and effectively enhanced the body’s mucosal defense function.

Acute low-temperature stress induces ROS and activates the antioxidant system [26]. The Keap1-Nrf2 signaling pathway plays a key role in antioxidant defense [27]. SOD, CAT, and GSH-Px are key enzymes involved in maintaining redox homeostasis [28]. MDA reflects the extent of oxidative damage [29]. In this study, acute low-temperature stress led to an increase in hepatic MDA levels and a decrease in SOD and CAT activity at 48 h, indicating impaired ROS scavenging capacity. This may be due to metabolic suppression, as well as elevated liver *nrf2* and *keap1a* mRNA levels, but impaired transcription of downstream genes (*sod* and *gsh-px*) [30,31]. In contrast, the H1 group significantly reduced liver MDA levels while simultaneously upregulating *nrf2*, *keap1a*, and *keap1b* mRNA levels, as well as the expression and enzymatic activity of downstream antioxidant genes. This suggests that HRW can directly scavenge ROS and activate the Keap1-Nrf2 pathway, effectively enhancing hepatic antioxidant capacity and preventing excessive oxidative stress responses [13]. In intestinal tissue, SOD activity in the control group peaked at 24 h, while CAT activity peaked at 48 h. This aligns with the cascade reaction of antioxidant enzymes: SOD acts as the first line of defense, converting O^2^^−^ into H_2_O_2_ and O_2_, followed by a delayed increase in CAT activity that catalyzes the decomposition of H_2_O_2_ into H_2_O and O_2_ [32]. The lack of significant changes in GSH-Px activity in the control group is attributed to the fact that the GSH-Px catalytic reaction consumes reduced glutathione (GSH). Under conditions of limited energy supply caused by acute hypothermia, the body may have prioritized CAT for the removal of peroxides [33]; meanwhile, the stable MDA levels indicate that the intestine possesses strong homeostatic repair capabilities. HRW exhibited a more rapid protective effect in the intestine compared to the control group. In the early stages of stress, CAT and GSH-Px activities in the H1 group increased significantly, alleviating stress by accelerating the clearance of peroxides and reducing MDA levels to below pre-stress levels by 48 h. This indicates that HRW enhanced the intestine’s ability to restore redox homeostasis. It is worth noting that H1 group also exhibited an overall downregulation of antioxidant gene expression associated with the Keap1-Nrf2 pathway. This may be attributed to hydrogen directly penetrating the intestinal epithelium to neutralize harmful •OH radicals and scavenge ROS [13], thereby reducing the activation of this pathway and redirecting more ATP to metabolic core organs such as the liver. As respiratory organs directly exposed to the environment, gills are particularly sensitive to environmental changes [34,35]. Acute low-temperature stress inhibits Na^+^/K^+^-ATPase activity, disrupting ionic balance and osmotic stability [36]. In this study, SOD activity in gill tissue of the control group significantly decreased at 8 and 24 h post-stress, leading to a significant increase in MDA content at 24 h, indicating lipid peroxidation in the gill tissue. By 48 h, both MDA and SOD activity had returned to baseline levels, suggesting that after acute low-temperature stress, the gills may maintain redox homeostasis through spontaneous regulation of antioxidant gene expression (increased *cat* mRNA levels) [5], although the protective effect is limited. In contrast, HRW activated the Keap1-Nrf2 signaling pathway for a defensive response by downregulating *keap1a* and *keap1b* and upregulating *nrf2* mRNA levels, thereby maintaining membrane stability [37]. However, the H3 group did not exhibit further antioxidant synergistic effects. This may be related to the dose–response relationship of hydrogen: low concentrations of hydrogen are effective as selective antioxidants, while excessively high concentrations may cause hydrogen to dissipate too rapidly due to saturation.

The intestinal microbiota is crucial for maintaining intestinal homeostasis and is highly sensitive to temperature fluctuations [38,39]. Acute low-temperature stress affects both the diversity and function of intestinal microorganisms [40]. This study found no significant differences in alpha diversity indices (Chao1, Shannon, and Simpson) among groups under acute low temperature. However, the Ace index and number of unique OTUs significantly increased in the H3 group, suggesting that high-concentration HRW may have enhanced the functional redundancy of the microbial community and promoted the colonization of endemic microorganisms. High-concentration HRW may enhance microbial richness, helping fish establish a more stable microbial barrier and strengthen intestinal barrier function.

In this study, the dominant phyla at the phylum level were *Pseudomonadota*, *Actinomycetota*, and *Bacillota*, consistent with previous research findings [41]. HRW (particularly in the H1 group) significantly optimized the microbial community structure by enhancing the relative abundance of *Bacillota*, which produces short-chain fatty acids (SCFAs), and reducing that of *Pseudomonadota*, which contains conditionally pathogenic bacteria, thereby alleviating intestinal metabolic and inflammatory [42]. Additionally, *Actinomycetota* suppressed pathogens by producing antibiotics [43]. This was confirmed at the genus level. The H1 group significantly enriched *Cetobacterium* and *Clostridium*; the former synthesizes vitamin B_12_ and promotes carbohydrate metabolism [44], while the latter generates SCFAs that energize the intestinal mucosa [45]. Simultaneously, the H2 and H3 groups exhibited significantly reduced relative abundance of the conditionally pathogenic bacterium *Acinetobacter*. Moreover, the H3 group showed the emergence of *Akkermansia*, which possesses probiotic potential, consistent with previous studies [46,47]. This suggests that different concentrations of HRW exert differential regulatory effects on the intestinal microbiota. In summary, the beneficial transformation of intestinal microbial community structure helps reduce dysbiosis-induced inflammation and improve intestinal health. This aligns with histopathological observations of improved intestinal architecture, further confirming HRW’s mitigating effect on intestinal injury. This study provides only preliminary insights into the modulatory effects of HRW on the intestinal microbiota in terms of alpha diversity and community composition; it does not delve into the statistical significance of differences in microbial structure or identify specific indicator species. Further validation using metagenomics and metabolomics is still required [48].

Although this study demonstrated that HRW has significant potential for alleviating acute low-temperature stress in fish, its large-scale application requires a comprehensive assessment of economic feasibility and technical adaptability [49]. The HRW preparation method used in this study is cost-effective and technically mature. However, scaling it up to commercial ponds would present challenges such as ensuring a continuous water supply and maintaining concentration levels. Future research should evaluate dose–response relationships across different aquaculture systems, develop automated delivery systems, and conduct life-cycle economic analyses to identify key factors for commercialization. Overall, as a green, residue-free antioxidant, HRW holds broad application prospects in the aquaculture sector, where extreme weather events are becoming increasingly frequent; however, further techno-economic evaluations are needed to translate laboratory findings into practical applications.

## 5. Conclusions

The results of this study indicate that the H1 group significantly improved the growth performance of juvenile largemouth bass. Acute low-temperature stress caused pathological symptoms in the livers of all groups, including hemorrhage, hepatocyte edema, vacuolization, and nuclear displacement, as well as intestinal tissue damage. The H1 group effectively alleviated these pathological symptoms. The H1 group significantly increased the enzymatic activities of SOD, CAT, and GSH-Px in the liver, as well as CAT and SOD in the intestine and gills, while significantly reducing MDA levels, thereby enhancing the antioxidant capacity of largemouth bass. Gene expression results showed that HRW significantly upregulated the expression of antioxidant genes (*sod*, *cat*, and *gsh-px*) in the liver and gills while downregulating these genes in the intestine. 16S rDNA analysis revealed that HRW increased the diversity of the intestinal microbiota and significantly enhanced the relative abundance of *Bacillota*. Notably, the protective effects of HRW did not increase linearly with rising concentrations, suggesting the existence of an optimal effective concentration (H1 group).

## Figures and Tables

**Table 1:** qPCR primer sequences.

Genes	Primer Sequences	Accession No.
*β-actin*	F: CACACAGTGCCCATCTATGA R: TGAAGCTGTAACCCCTCTCAG	MH018565.1
*keap1a*	F: CAGGTGGTGGGAAGACTTATTG R: AAGCGAGCGATGCCGAT	XM_038728593.1
*keap1b*	F: CAGCATTACATGGCCGCATC R: CTTCTCTGGGTCGTAAGACTCC	XM_038713667.1
*nrf2*	F: CAGACAGTTCCTTTGCAGGC R: AGGGACAAAAGCTCCATCCA	MW465398.1
*gsh-px*	F: CCCTGCAATCAGTTTGGACA R: TTGGTTCAAAGCCATTCCCT	MK614713.1
*sod*	F: CCACCAGAGGTCTCACAGCA R: CCACTGAACCGAAGAAGGACT	XM_038713339.1
*cat*	F: GTTCCCGTCCTTCATCCACT R: CAGGCTCCAGAAGTCCCACA	MK614708.1

**Table 2:** Effects of HRW on growth performance of largemouth bass.

Group	FBW (g)	SGR (%)	HSI (%)	CF (%)	SR (%)
Control	8.20 ± 0.42 ^b^	3.96 ± 0.12 ^b^	3.80 ± 0.28 ^a^	1.83 ± 0.07 ^b^	98.89 ± 0.28 ^a^
H1	12.60 ± 0.95 ^a^	5.51 ± 0.18 ^a^	3.69 ± 0.42 ^a^	2.13 ± 0.11 ^a^	98.06 ± 0.73 ^a^
H2	8.80 ± 0.74 ^b^	4.19 ± 0.22 ^b^	4.25 ± 0.12 ^a^	1.94 ± 0.05 ^ab^	97.78 ± 0.73 ^a^
H3	9.20 ± 0.48 ^b^	4.34 ± 0.23 ^b^	4.30 ± 0.44 ^a^	2.09 ± 0.05 ^a^	98.89 ± 0.28 ^a^

Note: Different lowercase letters indicate significant differences between groups (*p* < 0.05).

**Table 3:** Effects of HRW on intestinal villus height during acute low temperature in largemouth bass.

Group		Time (h)			
		0	8	24	48
VH (μm)	Control	492 ± 20 ^Aa^	493 ± 20 ^Aa^	411 ± 17 ^Bc^	361 ± 21 ^Bb^
	H1	489 ± 27 ^Aa^	378 ± 19 ^Bb^	497 ± 14 ^Ab^	457 ± 22 ^Aa^
	H2	458 ± 18 ^Ba^	358 ± 15 ^Cb^	572 ± 22 ^Aa^	462 ± 20 ^Ba^
	H3	489 ± 19 ^ABa^	411 ± 17 ^Cb^	498 ± 23 ^Ab^	435 ± 23 ^BCa^

Note: Different capital letters indicate significant differences among groups at different time points (*p* < 0.05); Different lowercase letters indicate significant differences between groups at the same time point (*p* < 0.05).

**Table 4:** Effect of HRW on the Alpha diversity index of largemouth bass at 24 h of acute low temperature.

Group	Ace	Chao1	Shannon	Simpson
Control	203.47 ± 37.40 ^b^	253.27 ± 58.82	5.11 ± 0.23	0.94 ± 0.01
H1	346.07 ± 18.49 ^ab^	342.76 ± 16.12	4.61 ± 0.06	0.89 ± 0.01
H2	208.15 ± 59.76 ^b^	206.37 ± 58.97	4.48 ± 0.43	0.88 ± 0.03
H3	408.20 ± 91.49 ^a^	330.66 ± 92.33	5.19 ± 0.30	0.93 ± 0.01

Note: Different lowercase letters indicate significant differences between groups (*p* < 0.05).

## Data Availability

The data supporting the findings of this study are openly available in the NCBI Sequence Read Archive (SRA) at https://dataview.ncbi.nlm.nih.gov/object/PRJNA1467364 (accessed on 19 May 2026), accession number PRJNA1467364. Data will be made available on request.

## Abbreviations

The following abbreviations are used in this manuscript:

HRW	hydrogen-rich water
ROS	reactive oxygen species
FBW	final body weight
SGR	specific growth rate
HSI	hepatopancreas somatic index
CF	condition factor
SR	survival rate
ALT	alanine aminotransferase
AST	aspartate aminotransferase
MDA	malondialdehyde
SOD	superoxide dismutase
CAT	catalase
GSH-Px	glutathione peroxidase
*nrf2*	nuclear factor erythroid 2-related factor 2
*keap1a*	kelch-1ike ECH-associated protein 1a
*keap1b*	kelch-1ike ECH-associated protein 1b
*cat*	catalase
*sod*	superoxide dismutase
*gsh-px*	glutathione peroxidase
HS	hepatic sinusoid
HE	hemorrhage
ICI	inflammatory cell infiltration
H	hepatocyte
PN	cellular peripheral nucleus
HD	hydropic degeneration
HPV	hepatocellular vacuolation
VH	villi length
VW	villi width
GC	goblet cell
MT	muscular thickness
SCFAs	short-chain fatty acids
GSH	glutathione reduced
